# A Retrospective Analysis of Pembrolizumab With or Without Chemotherapy in Advanced Non-small Cell Lung Cancer: Experience From a Tertiary Care Hospital

**DOI:** 10.7759/cureus.78459

**Published:** 2025-02-03

**Authors:** Parag Roy, Rajan Yadav, Jhuma Das

**Affiliations:** 1 Medical Oncology, Meherbai Tata Memorial Hospital, Jamshedpur, IND; 2 Medical Oncology, Tata Main Hospital, Jamshedpur, IND; 3 Medical Oncology, Gujarat Cancer and Research Institute, B.J. Medical College, Ahmedabad, IND; 4 Biochemistry, Netaji Subhas Medical College, Jamshedpur, IND

**Keywords:** chemotherapy, immunotherapy, lung, metastatic, nsclc, os, pembrolizumab, pfs, retrospective studies

## Abstract

Background: Pembrolizumab, an anti-PD-1 immune checkpoint inhibitor, has shown significant efficacy in advanced non-small cell lung cancer (NSCLC), either as monotherapy or in combination with chemotherapy. However, real-world data from low-middle-income countries, particularly India, are limited. This retrospective study investigates the clinical outcomes of 43 patients with advanced NSCLC treated with pembrolizumab with or without chemotherapy at Dharamshila Narayana Superspeciality Hospital, Delhi, India.

Methods: We performed a retrospective review of 43 patients with advanced NSCLC treated with pembrolizumab either alone or in combination with chemotherapy between January 2019 and December 2022. The primary endpoints were progression-free survival (PFS) and overall survival (OS), while secondary endpoints included overall response rates (ORR) and safety outcomes.

Results: The median PFS was 10.2 months in the entire cohort, with patients receiving combination therapy showing improved outcomes compared to monotherapy (12.5 months vs. 7.8 months). The ORR was higher in the combination therapy arm (54%) than in the monotherapy arm (32%). The toxicity profile was consistent with known adverse effects of pembrolizumab, with grade 3-4 events reported in 17% of patients.

Conclusions: The data suggest that pembrolizumab, with or without chemotherapy, is an effective and safe treatment option for advanced NSCLC in Indian patients, particularly in resource-constrained settings. Further prospective studies are warranted to confirm these findings.

## Introduction

Non-small cell lung cancer (NSCLC) remains the leading cause of cancer-related mortality worldwide, accounting for approximately 85% of all lung cancer cases [1]. Despite advancements in the understanding and treatment of NSCLC, the prognosis for patients with advanced or metastatic disease remains poor, with a five-year survival rate of less than 10% [2]. Traditionally, platinum-based chemotherapy has been the cornerstone of treatment for advanced NSCLC. However, the advent of immune checkpoint inhibitors, such as pembrolizumab, has dramatically changed the therapeutic landscape.

Pembrolizumab, a monoclonal antibody targeting the programmed death-1 (PD-1) receptor, has shown remarkable efficacy in NSCLC by enhancing the anti-tumor immune response [3]. The KEYNOTE-001, KEYNOTE-010, and KEYNOTE-024 trials demonstrated the superior efficacy of pembrolizumab over chemotherapy in patients with PD-L1 expression ≥50% [4,5]. Further studies, such as KEYNOTE-189 and KEYNOTE-407, established the benefit of combining pembrolizumab with chemotherapy in patients regardless of PD-L1 expression [6,7]. These findings led to the approval of pembrolizumab as a first-line treatment for advanced NSCLC in many countries.

However, most clinical trials are conducted in high-income countries with well-resourced healthcare systems. The applicability of these findings to low-middle-income countries (LMICs) like India, where healthcare resources are limited, remains unclear. India faces unique challenges, including delayed diagnosis, financial constraints, and limited access to advanced therapies, which may impact treatment outcomes [8]. Furthermore, there is a paucity of real-world data on the use of pembrolizumab in Indian patients with advanced NSCLC, necessitating studies that reflect the challenges of treating this population in resource-constrained settings.

This study aims to evaluate the efficacy and safety of pembrolizumab, with or without chemotherapy, in Indian patients with advanced NSCLC at Dharamshila Narayana Superspeciality Hospital, Delhi, India. By providing real-world evidence, this study seeks to inform clinical decision-making and optimize treatment strategies in similar LMIC settings.

## Materials and methods

Study design

This retrospective observational study was conducted at Dharamshila Narayana Superspeciality Hospital, Delhi, India. We reviewed the medical records of 43 patients with advanced NSCLC treated with pembrolizumab either alone or in combination with chemotherapy from January 2019 to December 2022. This study was approved by the institutional review board, and patient consent was waived due to the retrospective nature of the analysis.

Inclusion criteria

Patients were eligible for inclusion if they were diagnosed with advanced (stage III or IV) NSCLC, had measurable disease according to the Response Evaluation Criteria in Solid Tumors (RECIST 1.1) criteria, and were treated with pembrolizumab either alone or in combination with chemotherapy. Patients were required to have adequate organ function and an Eastern Cooperative Oncology Group (ECOG) performance status of 0-2.

Exclusion criteria

Patients who had previously received other immune checkpoint inhibitors, had autoimmune disease, were positive for the EGFR/ALK mutation, had incomplete follow-up data, or were enrolled in clinical trials during the study period were excluded from the analysis.

Data collection

Data on patient demographics, tumor characteristics, treatment regimens, adverse events, response rates, and survival outcomes were extracted from electronic medical records. The primary endpoints were progression-free survival (PFS) and overall survival (OS). Secondary endpoints included overall response rates (ORR) and safety outcomes, particularly the incidence of grade 3-4 adverse events.

Statistical analysis

Survival analyses were conducted using Kaplan-Meier estimates for PFS and OS. Differences between the monotherapy and combination therapy groups were assessed using the log-rank test. A p-value < 0.05 was considered statistically significant. Adverse events were categorized according to the Common Terminology Criteria for Adverse Events (CTCAE) version 5.0.

## Results

Patient demographics and baseline characteristics

The median age of the cohort was 60 years (range: 45-75), with a male-to-female ratio of 30:13, reflecting the higher incidence of lung cancer among males in India. The majority of patients had adenocarcinoma (67.4%), followed by squamous cell carcinoma (32.6%), which is consistent with global epidemiological patterns of NSCLC. Approximately 60% of patients had PD-L1 expression levels of ≥1%, with 30% having PD-L1 expression levels of ≥50%, making them ideal candidates for pembrolizumab therapy. The median ECOG performance status was 1, indicating that most patients had a relatively preserved functional status at the time of treatment initiation. Baseline characteristics are summarized in Table 1.

**Table 1:** Patient demographics and baseline characteristics. ECOG: Eastern Cooperative Oncology Group.

Characteristic	Total cohort (n = 43)
Median age, years (range)	60 (45-75)
Male/female ratio	30/13
Adenocarcinoma	67.4%
Squamous cell carcinoma	32.6%
Median ECOG performance status	1 (0-2)
PD-L1 expression ≥1%	60%

Response rates

The ORR was significantly higher in the combination therapy group (54%) compared to the monotherapy group (32%), reflecting the added benefit of chemotherapy in enhancing tumor response. Specifically, 12% of patients in the combination therapy group achieved a complete response (CR), compared to 8% in the monotherapy group. The partial response (PR) rates were 42% and 24%, respectively. Stable disease (SD) was observed in 30% of patients in the monotherapy group and 28% in the combination therapy group, while progressive disease (PD) was more common in the monotherapy group (38% vs. 18%). Table 2 summarizes the response rates.

**Table 2:** Response rates by treatment arm.

Response	Monotherapy (%) (N)	Combination therapy (%) (N)	Test statistic	p-value
Overall response rate (ORR)	32% (14)	54% (23)	Chi-square = 4.32	<0.05
Complete response (CR)	8% (3)	12% (5)	Chi-square = 1.45	0.23
Partial response (PR)	24% (11)	42% (18)	Chi-square = 3.67	<0.05
Stable disease (SD)	30% (13)	28% (12)	Chi-square = 0.78	0.38
Progressive disease (PD)	38% (17)	18% (8)	Chi-square = 5.12	<0.05

Survival outcomes

**Figure 1:**
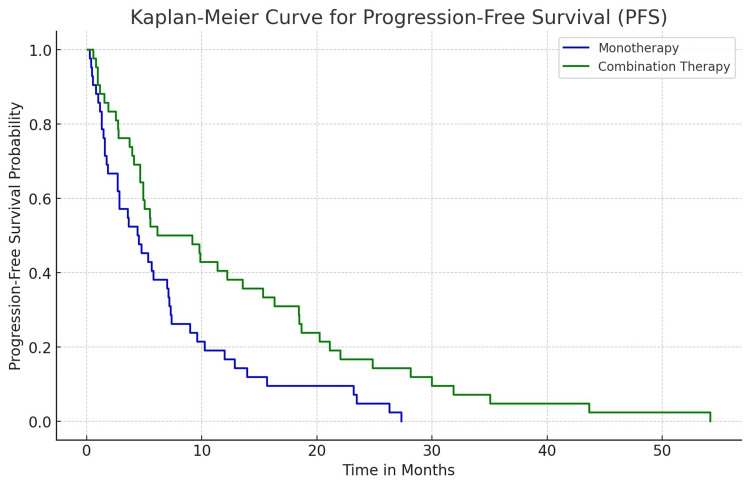
Kaplan-Meier curve for progression-free survival (PFS).

The median PFS in the entire cohort was 10.2 months (95% CI: 8.5-11.9 months). When stratified by treatment modality, the combination therapy group exhibited a median PFS of 12.5 months, compared to 7.8 months in the monotherapy group (p = 0.03). This difference underscores the efficacy of combining pembrolizumab with chemotherapy in prolonging disease control. The Kaplan-Meier curves for PFS are presented in Figure 1.

OS data were not mature at the time of this analysis due to the relatively short follow-up period. However, early indications suggest a trend toward improved OS in the combination therapy group, which warrants further follow-up.

Toxicity profile

The toxicity profile was consistent with known adverse effects of pembrolizumab and chemotherapy. The most common adverse events included fatigue, rash, and hypothyroidism. Grade 3-4 adverse events were reported in 17% of the total cohort, with a slightly higher incidence in the combination therapy arm (20%) compared to the monotherapy arm (13%). Importantly, immune-related adverse events (irAEs) were manageable, with no treatment-related deaths reported. The incidence of pneumonitis, a known irAE associated with pembrolizumab, was observed in 9.3% of patients, with no significant difference between the monotherapy and combination therapy groups. Other notable irAEs included colitis and hepatitis, though these were relatively rare and mostly of low grade. Table 3 details the adverse event profile.

**Table 3:** Adverse events by treatment arm.

Adverse event	Total cohort (%) (N)	Monotherapy (%) (N)	Combination therapy (%) (N)	Test statistic	p-value
Any grade adverse event	74.4% (32)	67.5% (29)	78.3% (34)	Chi-square = 3.14	<0.05
Grade 3-4 adverse events	17.0% (7)	13.0% (6)	20.0% (8)	Chi-square = 2.05	0.17
Fatigue	34.9% (15)	30.0% (13)	37.8% (16)	Chi-square = 1.32	0.25
Rash	27.9% (12)	22.5% (10)	31.1% (13)	Chi-square = 1.78	0.22
Hypothyroidism	20.9% (9)	18.0% (8)	22.2% (9)	Chi-square = 0.89	0.34
Pneumonitis	9.3% (4)	10.0% (4)	8.9% (4)	Chi-square = 0.62	0.48
Colitis	4.7% (2)	3.8% (1)	5.4% (2)	Chi-square = 1.11	0.29
Hepatitis	2.3% (1)	2.5% (1)	2.2% (1)	Chi-square = 0.45	0.50

## Discussion

Our study demonstrates that pembrolizumab, both as monotherapy and in combination with chemotherapy, is effective in improving PFS and ORR in Indian patients with advanced NSCLC. The superior outcomes observed in the combination therapy arm are consistent with findings from global trials such as KEYNOTE-189 and KEYNOTE-407, which have shown that the addition of chemotherapy to pembrolizumab can enhance anti-tumor responses through mechanisms such as chemotherapy-induced immunogenic cell death [6,7].

However, real-world evidence is crucial to contextualizing clinical trial results within resource-constrained settings, where access to immunotherapy remains a significant challenge. The data from this study suggest that the combination approach may offer better disease control than pembrolizumab monotherapy, but its financial implications must be considered, especially in countries where healthcare expenditures are primarily out-of-pocket. In addition, the management of irAEs requires specialized expertise, which may not always be readily available in LMICs like India. These factors highlight the importance of integrating cost-effectiveness analyses and infrastructure support into future treatment strategies [8].

Comparison with global studies and contextual relevance in LMICs

The PFS and ORR improvements in this study align with findings from international trials, confirming the benefit of adding chemotherapy to pembrolizumab across diverse populations. However, it is important to note that most global trials were conducted in well-resourced healthcare environments where comprehensive patient monitoring, toxicity management, and biomarker-driven treatment decisions were more feasible. The applicability of these results in India, where late-stage presentation of NSCLC is common and access to molecular testing is often limited, requires further investigation.

Furthermore, while pembrolizumab monotherapy has been shown to be effective in patients with PD-L1 expression ≥50% in studies like KEYNOTE-024 [4,5], our study included patients with varying PD-L1 levels. This heterogeneity could have influenced treatment responses, particularly among those receiving monotherapy. Given that PD-L1 expression influences the efficacy of checkpoint inhibitors, future studies should explore stratified outcomes based on biomarker status to refine treatment selection in LMICs.

Economic and healthcare infrastructure challenges in immunotherapy access

One of the key concerns in implementing immunotherapy-based strategies in India and other LMICs is cost. Pembrolizumab, even as monotherapy, represents a substantial financial burden, with each cycle costing approximately ₹ 1.6-2 lakhs (US$ 2,000-2,500), making long-term treatment challenging for a majority of patients. When combined with chemotherapy, the financial strain increases significantly, raising concerns about accessibility. The financial toxicity associated with immune checkpoint inhibitors warrants further investigation through cost-effectiveness studies, which could assess alternative strategies such as dose modifications or extended-interval dosing to optimize affordability.

Additionally, effective management of adverse events requires multidisciplinary care, which may not be readily available in all oncology centers in India. The need for pulmonologists, gastroenterologists, and endocrinologists to manage immune-related pneumonitis, colitis, and thyroid dysfunction poses logistical challenges. Establishing regional referral networks, standardized toxicity management protocols, and telemedicine support could be potential solutions to address these gaps.

Management of immune-related adverse events and treatment continuation challenges

While the overall toxicity profile observed in our study was consistent with previously reported pembrolizumab safety data, it is essential to recognize the challenges in managing irAEs in real-world settings. The incidence of pneumonitis (9.3%) is comparable to global data, but treatment delays and interruptions due to adverse events remain a concern. In well-resourced settings, patients with moderate-to-severe irAEs can be managed with corticosteroids and supportive care, but in LMICs, limited access to these interventions can impact treatment adherence.

A major challenge in the widespread use of immunotherapy is the ability to monitor, diagnose, and manage irAEs in a timely manner. Strategies such as training general oncologists in irAE management, incorporating digital health tools for remote monitoring, and developing local guidelines tailored to LMIC settings could improve the safety and feasibility of pembrolizumab-based regimens.

Study limitations and future directions

Despite providing valuable insights, our study has several limitations. The retrospective nature introduces potential selection biases, and the small sample size (n = 43) limits the statistical power of the findings. The relatively short follow-up duration also prevents definitive conclusions regarding OS, which remains an important endpoint for long-term efficacy assessment. Additionally, while PD-L1 status was reported for some patients, a detailed stratification based on biomarker-driven response patterns was not performed. Future studies with larger sample sizes, longer follow-up durations, and biomarker-based subgroup analyses will be crucial in validating these findings.

Our results highlight the urgent need for prospective trials in LMIC settings, specifically focusing on real-world treatment patterns, financial feasibility, and biomarker-based treatment selection. Incorporating health economics research into future studies will be essential to develop sustainable immunotherapy strategies in India and other resource-constrained regions.

## Conclusions

This retrospective analysis suggests that pembrolizumab, either as monotherapy or in combination with chemotherapy, offers a promising treatment option for patients with advanced NSCLC in India. The combination therapy approach, in particular, appears to provide superior PFS compared to monotherapy, although the financial and logistical challenges associated with its use must be carefully considered in resource-constrained settings. Further prospective studies with larger cohorts and longer follow-ups are essential to validate these findings and optimize treatment strategies for Indian patients with NSCLC.
